# Nurturing virtues of the medical profession: does it enhance medical students’ empathy?

**DOI:** 10.5116/ijme.5951.6044

**Published:** 2017-07-11

**Authors:** Marcelo Schweller, Diego Lima Ribeiro, Eloisa Valer Celeri, Marco Antonio de Carvalho-Filho

**Affiliations:** 1 Department of Emergency Medicine, Faculty of Medical Sciences, University of Campinas, Brazil; 2 Department of Psychiatry, Faculty of Medical Sciences, University of Campinas, Brazil

**Keywords:** Empathy, professional identity, medical education

## Abstract

**Objectives:**

To examine if the empathy levels of first-year medical students are amenable to didactic interventions idealized to promote values inherent to medical professional identity.

**Methods:**

This is a pretest-posttest study designed to assess the empathy levels of first-year medical students (n=166) comprising two consecutive classes of a Brazilian medical school, performed before and after a didactic intervention. Students attended a course based on values and virtues related to medical professional identity once a week over four months. Every didactic approach (interviews with patients and physicians, supervised visits to the hospital, and discussion of videotaped simulated consultations) was based on “real-world” situations and designed to promote awareness of the process of socialization. Students filled out the Jefferson Scale of Physician Empathy (JSPE) on the first and last days of this course, and the pretest-posttest analysis was performed using the Wilcoxon Signed Rank Test.

**Results:**

The mean pretest JSPE score was 117.9 (minimum 92, maximum 135) and increased to 121.3 after the intervention (minimum 101, maximum 137). The difference was significant (z=-5.2, p<.001.), with an effect size of 0.40. The observed increase was greater among students with lower initial JSPE scores.

**Conclusions:**

Empathy is a fundamental tool used to achieve a successful physician-patient relationship, and it seems to permeate other virtues of a good physician. This study’s results suggest that medical students’ empathy may be amenable to early curricular interventions designed to promote a positive development of their professional identity, even when empathy is not central in discussion.

## Introduction

Throughout history, the practice of medicine has been characterized by committed physicians who were satisfied with their profession, and this has always been recognized and admired by patients.^1^ Recent decades have witnessed many changes in the way we practice medicine, and although there are still many physicians who are proud of their profession and patients who are proud of their physicians, this happy group of people is probably shrinking. Unfortunately, this process starts before a physician’s professional practice itself, during undergraduate medical education, when medical students’ empathy levels may begin to decrease.^2^^,^^3^  This process may affect the consolidation of the professional identity, which results in physicians not truly committed to patients’ interests.^4^ At first, medical students are often full of dreams and expectations, and are inspired by the prospect of self-development and contribution to the benefit of the community.^5^ However, medical school is a period of intense contact with disease, suffering and death, which often occurs at an age when students have had little personal experience with these issues and few opportunities to reflect on them.

To address this challenge in a positive way, it would be essential for students to have a teacher who has reflected on these things and who realizes the meaning of the commitment necessary to become a good physician. However, teachers themselves may be dissatisfied with their profession, and may eventually convey to students the impression that they should be less personally involved with the issues raised by their patients in their daily practice. Negative role models may undermine students' willingness to overcome the challenges related to patient care.^4^

Not infrequently, students are confused by these negative role models, and question the motivations that led to their career choice, which ends up distorting the way they see the practice of medicine. These negative experiences throughout the medical program interfere adversely with students’ capacity for being empathetic and developing genuine bonds.

This process directly affects patients. Empathy is one of the concepts related to medical identity, and it has been shown that physicians’ empathic behavior is associated with patient satisfaction,^6^^,^^7^ adherence to treatment^7^^,^^8^ and treatment outcomes.^9^^,^^10^ A recent study showed that patients with deep relationships with their general practitioners discussed more problems and issues during consultations.^11^ This process is also harmful to doctors, who lose sight of the meaning of their day-to-day practice and feel frustrated about having moved away from the practice of medicine in which they too once believed. In this context, a defense mechanism may emerge in reaction to an environment perceived as hostile, culminating in the development of a cynical and even arrogant attitude.^12^

It is not yet clear in the medical education literature which strategies are best employed to address the negative effects of the hidden curriculum in the process of professional identity formation during the undergraduate medical course. We developed a mandatory curriculum for first-year medical students in a Brazilian university, aiming to discuss and reflect on the hidden curriculum and on the role of the doctor in the doctor-patient relationship. The rotation was based on didactic interventions with real-world appeal, using several approaches to share with students the importance of being aware of the process of socialization and professional identity formation.

Professional identity is based on the consolidation of values and virtues inherent to the practice of medicine. Many of these virtues depend on the physician’s capacity to put him or herself in another person’s shoes, i.e., to be empathic. In this sense, empathy may be a tool for someone to be a virtuous physician. Our hypothesis is that discussing this matter with students from the beginning of their medical training, reflecting on future challenges they may face and providing them with the tools with which they may cope may benefit their formative process.

The purpose of this study is to examine if empathy, a concept related to medical identity, may be amenable to this type of early curricular intervention.

## Methods

### Study design and participants

This is a pretest-posttest study designed to assess the empathy levels of first-year medical students (n=166) comprising two consecutive classes (2012 and 2013) of a medical school in Brazil, performed before and after they participated in a curricular course called Health and Medicine (H&M). This study was approved by the Research Ethics Committee in Human Beings of the Faculty of Medical Sciences at the State University of Campinas (Unicamp).

Through a partnership between the department of Psychiatry and the discipline of Emergency Medicine, this course presents the process of professional identity formation in a positive way, reinforcing the meaning of becoming a doctor and the fulfillment we can achieve in our everyday lives as physicians.

Every issue, including the suffering and difficulties encountered by patients and physicians, was discussed based on real situations. Our idea was to show that sharing someone’s pain is not necessarily painful. Moreover, contact with the experiences of our patients invites us to reflect on ourselves, which invariably leads to personal growth and development.

This course lasted four months for the classes of 2012 and 2013 and was composed of various formats of didactic interventions, which are described below.

### Interviews with physicians

In the first meetings, students heard testimony from professors representing different medical specialties (emergency medicine, internal medicine, oncology, surgery, pediatrics, OB&Gyn) who are notoriously satisfied with their profession. In these meetings the professors shared with the students some of their life stories. These included the professors’ paths in the medical profession from the moment they chose medicine as their career, through the difficulties encountered on the way and how they handled them. Students asked questions freely and were able to understand the meaning of the medical profession to these professionals. The sessions were often intense and emotional and lasted up to two hours.

### Interviews with patients

Although there are reports of the direct participation of patients in medical education at other institutions, our school had not yet taken that step in a systematic way. We invited patients undergoing care at our teaching hospital and clinic to join us in the education of our medical students.

To this end, we instructed participating patients to tell their stories, emphasizing their experiences with health services (especially with their physicians), and their perceptions of and feelings about their disease and about their doctors. We asked patients to share with students what type of behavior in physicians motivated or demotivated them to follow their therapeutic plans. The idea was to show students that, sometimes, physicians could be misunderstood or even seem rude, in spite of not meaning to.

We also asked patients to talk about the impact of disease in their lives. Students were able to notice that although disease brings fear and limitations, it can also be a trigger for reflection, and even an opportunity for change.

These interviews were conducted in the classroom by teachers and students themselves, to provide students a first contact with the realities of both doctor and patient issues.

### Supervised visits to the hospital

In pairs, students visited the emergency room, the intensive care unit, the internal medicine ward or the psychiatric emergency center of our hospital. They were supervised by selected physicians who openly enjoy being doctors, and followed them during actual calls. Each student had one supervised visit to the hospital.

In this activity, students were able to observe the actual function of these areas of the hospital, by participating in rounds, consultations, conversations with family members and interactions among members of the healthcare team. Whenever possible, instructors held discussions with patients or family members about the course of disease, including issues related to death and end of life care. After the service, doctors remained available to reflect on the sessions and answer questions from students.

Separately, the students went to the hospital by themselves to talk to patients who were waiting for consultations in the outpatient clinic. In these conversations, the students listened to the opinions of the patients and were able to understand the relationship of those patients with their physician and the health care system. After each visit to the hospital, supervised or not, students were asked to produce a reflexive writing assignment and to discuss their perceptions in a group of classmates and teachers.

### Videos of simulated consultations performed by teachers and actors

We displayed videotaped simulated consultations using a standardized patient (SP). One of the authors (MS) performed the role of the doctor in the same clinical situation three times, each time exhibiting a different attitude (a rude and unethical doctor, an unethical although polite doctor, and a good doctor).

In the first consultation, the doctor clearly placed himself above the patient, conducting the interview in a distant and even rude manner. Throughout the second consultation, the doctor behaved politely and followed some of the patient’s hints but still displayed a critical and judgmental attitude towards the patient. Finally, in the last consultation, the doctor displayed genuine empathic behavior, and only then did the patient feel comfortable sharing his concerns and describing the uncomfortable side effects that accompanied the use of the prescribed medication.

Students were able to observe and discuss the doctor-patient relationship and imagine the feelings of the patient in each consultation. They reported realizing that the attitude of the physician affects the outcome of the consultation because in some situations the patient does not feel comfortable sharing with the physician what is actually happening. In addition, we were able to demonstrate that the last consultation (the good one) was no longer than previous ones.

### Caricatures of “really bad” medical consultations

Finally, teachers and SPs participated in simulated situations in front of the audience of first-year students, portraying caricatures of inadequate medical consultations. In this activity, the authors (MS and MACF) intentionally behaved inappropriately in the role of the physician, by embodying some common negative clichés of the doctor-patient relationship, such as interrupting the patients’ speech, not following evident hints, blaming patients for their illnesses and being rude.

The SPs were instructed to stop the scene whenever they found it necessary to share with the audience what the patient thought and felt in response to some of the doctors’ actions. We encouraged actors to use sarcasm to ridicule the physicians’ behavior. Our intention was to show students that sometimes patients have a bad impression of physicians’ attitudes but do not always share them with the doctor.

The fun and laughter triggered by the scenes did not compromise the discussion held after the activity, in which the students shared with us their perceptions of the issues discussed and even personal stories in which they had experienced similar situations when they themselves or family members were patients.

### Data collection

On the first day of class, we welcomed the students and shared with them the purpose of the H&M course. Subsequently, we presented this study and distributed informed consent documents to those who decided to participate in this research. At the same time, the participants filled out the pretest of the student version of the Jefferson Scale of Physician Empathy (JSPE).

JSPE is a 20-item, 7-point Likert scale used to assess empathy specifically in the context of patient care, via self-report.13 In other words, it reflects the individual’s view of several characteristics of the doctor-patient relationship. Students should read each statement and verify their level of agreement from 1 (strongly disagree) to 7 (strongly agree). Some of the items are reverse scored, and the sum of the points from each item generates a total score of up to 140. A factor analysis of JSPE resulted in three distinct factors: (1) ‘perspective taking’, comprising 10 items; (2) ‘compassionate care’, comprising 8 items; and (3) ‘standing in the patient’s shoes’, comprising 2 items. This scale has been validated in various languages and has been used extensively in empathy research on graduate and post-graduate medical education. We used the Portuguese language version of the JSPE in our study.^14^

Throughout the semester, students participated in the didactic interventions described above. On the last day of class, they filled out the posttest of the JSPE (four months after the pretest application).

### Data analysis

Exploratory data analysis was performed through summary measures (mean, standard deviation, minimum, median and maximum). The comparison between pretest and posttest was achieved through the Wilcoxon Signed Rank test. The level of significance was 5%. We also used the effect size to infer the practical relevance of the findings. The computational program used was the SAS System for Windows version 9.2. SAS Institute Inc., 2002-2008, Cary, NC, USA.

## Results

### Empathy levels

**Figure 1:**
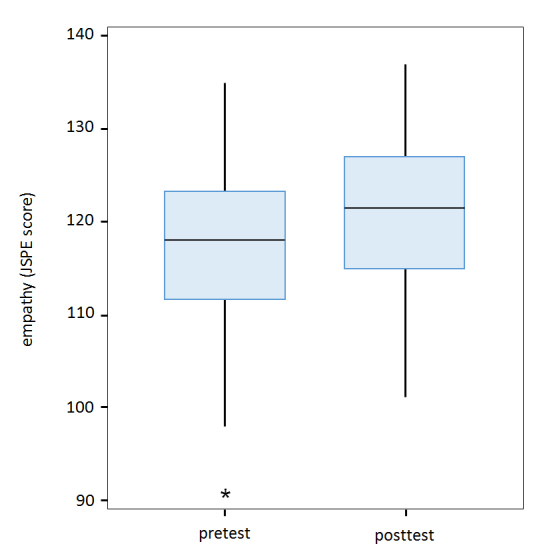
Boxplot of the empathy levels of 166 first-year medical students before and after the didactic intervention. Empathy was measured with the Jefferson Scale of Physician Empathy (JSPE).

The mean pretest JSPE empathy score of the first-year students was 117.9 (minimum = 92, maximum = 135, median = 118, SD = 8.7); the mean posttest score was 121.3 (minimum = 101, maximum = 137, median = 121.5, SD = 7.7, p<0.001).  The Wilcoxon Signed Rank Test, which evaluated difference between mean empathy scores before and after the intervention, is significant (z = -5.2, p < .001). The effect size (ES), a test to determine the power (or practical significance) of the result, was 0.40, which is considered relevant, although small. Figure 1 shows the mean pre- and posttest JSPE scores for these students.

With the intention of determining which students had the greatest variations in empathy levels after the activities, initial JSPE scores were divided into quartiles. We observed that the increase in empathy levels was significantly greater for those students that had a lower level of empathy before the activity. The boxplots in Figure 1 show that the minimum score was significantly higher at posttest.

### Student participation and teachers’ impressions

Teachers involved in the H&M course could not avoid observing that, as the course developed, students showed an increasing interest in subsequent class meetings. It became evident that the number of students who actively participated in class discussions was greater every week. Students came to us personally or via email to share personal difficulties or achievements, and some even asked to accompany us during our night shifts in the emergency room. Approaches involving SPs were also important to encourage student participation because, despite the seriousness of the issues discussed, they felt secure enough to share their opinions – even the embarrassing ones.

During discussions, we confirmed our idea that medical students enter the undergraduate program with very positive attitudes towards the practice of medicine. However, even in the first year of medical school some students already express questionable ideas regarding doctor-patient relationship, such as the unquestionable authority of the physician to control the consultation, the need to blame patients’ lifestyles as the causes of their illnesses, and threatening patients with complications that may arise if they do not follow their treatment properly.

Students also requested that the curriculum of the medical program could provide additional moments in which they could freely discuss the issues of the hidden curriculum and personal issues, among themselves and with more experienced individuals. Raising awareness of the process of professional identity formation empowered students to identify negative role modeling, which is important to enhance self-confidence and self-awareness.

## Discussion

Some studies have shown maintenance of^15^ or increases in^16^^-^^18^ medical students’ empathy levels during their undergraduate course. A recent review showed a trend toward higher empathy levels in later years of the undergraduate course,^19^ although another systematic review indicated that most studies on the subject found a decline in empathy,^20^ with hidden curriculum being the most reported cause of this phenomenon.^4^ This finding is in agreement with the students’ perception that the main causes for the decline in empathy are the lack of positive role models and time pressure.^21^

In our study, we observed that first-year medical students already show dissatisfaction with some aspects of their academic routines, particularly interpersonal relationships, and with the lack of adequate opportunities to discuss and reflect on these issues. In this way, it became clear that hidden curriculum starts from the first year of medical school and is not exclusive to the clinical course. Moreover, upon hearing some of these students’ ideas regarding the doctor-patient relationship, we wondered whether this process might begin even earlier.

Previous experiences as patients or when accompanying their relatives in consultations prior to entering medical school may have created a conception that is only enhanced by hidden curriculum. Moreover, different television series depict hidden curriculum in medicine, and sometimes even cynicism is evoked as a fundamental trait to medical competence. In this context, it could be difficult to realize the behaviors that could harm patients without proper guidance on reflection.^22^

We observed an increase in self-assessed empathy levels of first-year medical students after a series of targeted didactic interventions based on values and virtues of a good medical practice. We did not give “empathy lessons”; that is to say empathy was not the core topic in either of our discussions. Even so, it seems to permeate every aspect of the medical profession.

About half of medical students believe that empathy can be taught,^21^but there is no consensus on whether empathy is something that can be taught or learned. Some studies have demonstrated that targeted interventions may increase the empathy levels of medical students^23^ and residents, and their results were summarized in recent reviews.^24^^,^^25^ Other studies found no significant increases in empathy after brief interventions.^26^^,^^27^ There is also evidence that interventions performed sequentially may sustain the initial gain in empathy.^28^

We rely on the hypothesis that the first contact of medical students with the reality of the medical profession is crucial to the development of their professionalism, especially regarding the way they see the doctor-patient relationship. Highlighting the patients’ perspectives may also be an effective way to remind medical students to put themselves in their patients’ shoes.^29^ In this sense, a positive first experience within the medical curriculum may shield or buffer the influence of negative role models in the future.

Medical students react in a very personal way to the challenges of medical school, but it seems to us that many students bring concepts that are not originally theirs; rather, they belong to the models that they have encountered over the course of their studies, or even earlier influences, as we have speculated. One of these concepts is the need for personal distance from the patient with the purpose of self-protection, an attitude that frequently culminates in cynicism and loss of empathy. As students participated in the proposed interventions, we noticed that many felt more comfortable with the idea of the physician being closer to patients and acting more empathically in their daily practice. Students with lower initial empathy might have been those who were the most susceptible to the hidden curriculum and to negative role models and thus would have had the greatest gains in empathy after the interventions.

Initiating a good relationship with students in the first year of medical school is an opportunity for a longitudinal follow-up throughout the course. It would have the purpose of reinforcing ideas about professionalism and encouraging personal growth. Ideally, in each rotation, there would be at least one instructor committed to those ideas, who enjoys being a physician and, of course, a teacher.

### Implications for medical education and future studies

Empathy, or the ability to move from oneself to the other, is the initial step toward many of the fundamental virtues in medical practice. The hidden curriculum in medical school, embodied mainly by negative role models and work overload, may influence medical students early in their undergraduate course, sometimes directing them away from these virtues and toward cynicism and affective detachment. If the medical curriculum contemplates, from the beginning, issues related to the development of medical students’ professional identity (especially values and virtues) it may enhance students’ empathy and counterbalance the negative effects of the hidden curriculum.

Future studies may better understand and describe the relationship between empathy and the virtues required for medical practice, for example, using empathy scales and psychometric instruments that assess the moral behavior of physicians or medical students.

### Study limitations

Our study did not have a control group, narrowing the interpretation of the results. The observed increase in the empathy levels of our first-year medical students may have been overestimated due to the maturation bias (a natural process that leads participants to change over time) and due to the Hawthorne effect (the tendency for people to perform better when participating in an experiment and being observed). These phenomena may have led to higher posttest scores. 

In a similar way, the finding that the students with lower initial empathy levels had higher increases after the intervention may have been overestimated due to the phenomenon of regression to the mean (the tendency of low scores to increase and high scores to decrease on repeated testing) and the ceiling effect (those with higher scores have less room to improve).

Self-assessed empathy measures raise the question of whether their results really predict students’ actual behavior during real clinical practice, when they are interacting with patients. However, we believe that the answers applied to the scales used indicate at least the intention of being empathic, and the students’ perceptions of the importance of empathy in the doctor-patient relationship. It does not obviate the need to incorporate instruments to measure empathy considering the patients’ perspectives.^30^

### Acknowledgements

The authors would like to thank all the physicians, actors and patients who participated in the activities of this study with amazing dedication. Financial support for this study was received from the State of Sao Paulo Research Foundation (FAPESP) and the National Counsel of Technological and Scientific Development (CNPq).

### Conflict of Interest

The authors declare that they have no conflict of interest.
